# Microsatellite-Based Genetic Structure and Diversity of Local Arabian Sheep Breeds

**DOI:** 10.3389/fgene.2018.00408

**Published:** 2018-09-25

**Authors:** Raed M. Al-Atiyat, Riyadh S. Aljumaah, Mohammad A. Alshaikh, Alaeldein M. Abudabos

**Affiliations:** ^1^Animal Production Department, King Saud University, Riyadh, Saudi Arabia; ^2^Animal Production Department, Mutah University, Karak, Jordan

**Keywords:** ovis aries, gene flow, admixture, ancestry, biodiversity

## Abstract

The genetic diversity of the sheep breeds in the Arab countries might be considered to be a mirror of the ecology of the region. In this study, the genetic structure and diversity of sheep breeds from Saudi Arabia (Harri, Najdi, Naemi, Arb, and Rufidi) and Awassi sheep from Jordan as an out-group were investigated using 19 microsatellites. All the breeds had high intra-population genetic diversity expressed as allelic number (7.33) and richness (2.9) and, expected heterozygosity (0.77). Structure analysis revealed three main gene pools underlying the ancestral genetic diversity of the study populations. The first pool had Harri, Najdi, and Rufidi breeds; the second had Naemi and Awassi breeds, and the third had the Arb breed which was significantly differentiated from the other breeds. Factorial correspondence analysis lent further support to the presence of the three gene pools. Although the outgroup Awassi sheep was more clearly differentiated, it still genetically close to Naemi sheep. The differentiation of the Arb breed could have been resulted from geographic and reproductive isolation. On the other hand, the genetic structure of the other two gene pools could be the result of the past and recent gene flow between individuals reared in the region known to be the center for animal husbandry and trading until the current time.

## Introduction

The ecological diversity of the Arabian Peninsula has been reflected in the large number of sheep breeds found in the region (ACSAD (The Arab Center for the Studies of Arid Zones Dry lands)., 1997). The total numbers of breeds of sheep found in the Arab countries have been estimated to be between 46 and 49 indigenous breeds and are classified as fat-tailed, thin-tailed wool sheep and fat-tailed hairy sheep (FAO, 1995; ACSAD (The Arab Center for the Studies of Arid Zones Dry lands)., 1997). In fact, someone might find the three types of sheep in one country. For example, the Kingdom of Saudi Arabia (KSA) has six breeds of sheep named Harri (Habsi), Najdi, Naemi (Awassi), Arb, and Rufidi (ACSAD (The Arab Center for the Studies of Arid Zones Dry lands)., 2011; Aljumaah et al., 2014; Adam et al., 2015). Jordan, however, has only one indigenous breed of sheep named Awassi (Al-Atiyat et al., 2014), although sheep breeds, such as the Naemi and Najdi, from neighboring countries' were reported to be available in Jordan (Jawasreh et al., 2011). The Awassi has the widest geographic distribution of any sheep breed in the Arabian Peninsula; it is found in Saudi Arabia, Jordan, Palestine, Syria, Lebanon, Iraq, Turkey, and Egypt (Galal et al., 2008). In general, most sheep breeds in the Arabian Peninsula have been phenotypically described and characterized (ACSAD (The Arab Center for the Studies of Arid Zones Dry lands)., 2011). They have been and still are raised under either nomadic pastoral or transhumant production systems across the three geographic areas: the Horn of Africa, North Africa, and the Middle East (FAO, 1995). Some of the breeds are also raised under pastoral transhumance system but over a limited geographic range within a country. This is the current situation under which most breeds of sheep are reared in KSA. Given that the KSA shares land borders with Jordan, the likelihood of gene flow of Jordan Awassi sheep into KSA was reported (Rischkowsky and Pilling, 2007). It is also important to note that the KSA has been at the center of historical animal exchange networks following active ancient trade routes of Incense and Silk Roads (Christian, 2000). Currently, owners of the different sheep breeds in both countries have often faced major threats to their genetic diversity resulting from uncontrolled mating and other regional, climatic and global economic forces.

It is a common assumption that gene flow is influenced by landscape and topographical features of the regions (Taylor et al., 1993). Therefore, the sheep flock dynamics and gene flow within and between KSA regions might have shaped the genetic diversity of the breeds. Some recent studies have highlighted the genetic diversity of KSA sheep (Aljumaah et al., 2014; Adam et al., 2015). In addition, some studies have showed their differentiation from Jordan Awassi (Al-Atiyat and Aljumaah, 2014) and from Egypt and worldwide sheep (Peter et al., 2007). However, none of these studies provided information on the genetic structure of the local KSA sheep populations. It might be worthy to note that the genetic diversity is total amount of variation in a population, while population structure is how the variations are distributed and originated. Recently, Elbeltagy et al. (2015) reported that the genetic diversity and structure of Egyptian indigenous sheep reflects historical and recent anthropological interaction. There has been a suggestion that Saudi local domestic breeds have ancestors originating from within Saudi Arabia or nearby countries (Galal et al., 2008). The advent of molecular DNA technologies have provided great potential for investigating genetic diversity and structure as well as unravel the common genetic history of livestock populations. The aim of the present work was to investigate the genetic diversity, structure and common ancestry between sheep breeds found in the KSA through the analysis of genetic variation in microsatellite markers.

## Materials and methods

### Sheep populations

**Figure 1:**
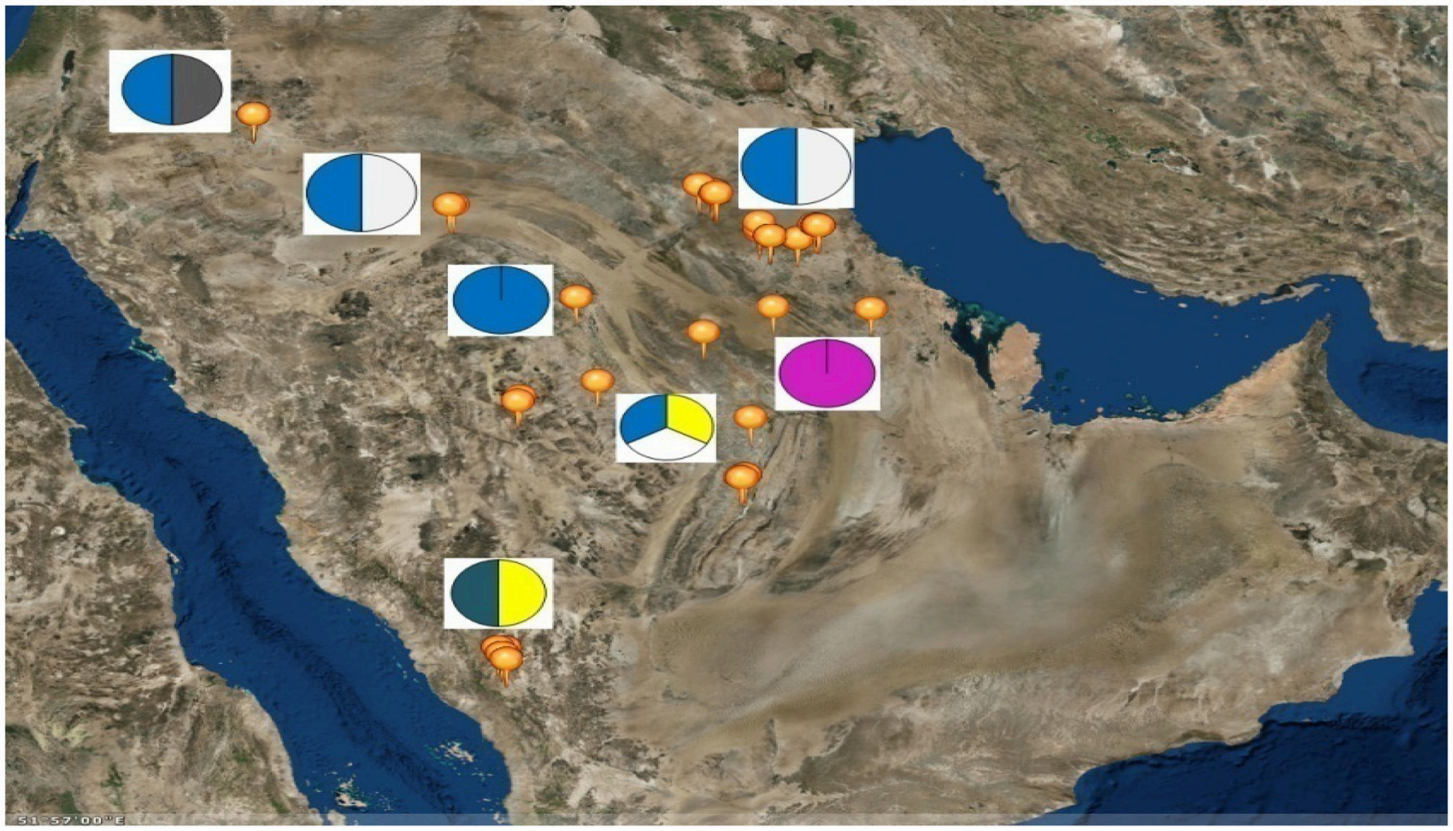
Map of the Kingdom of Saudi Arabia showing the geographical location of the six sampled sheep breeds as; Rufidi (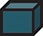), Harri (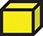), Najdi (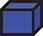), Naemi (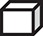), Awassi (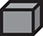), and Arb (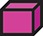).

Six sheep breeds/populations from different geographic regions in the KSA including South, North, and eastern parts were used in the present study (Figure 1). Blood samples were collected from unrelated adult males (rams) and females (ewes) of five KSA breeds; Harri (29), Najdi (31), Naemi (31), Arb (37), and Rufidi (6). In addition, 6 unrelated adult males of the Jordanian Awassi were also sampled and used in the study as an out-group breed. The rams were sampled at their farm limiting to two rams per farm per village or rural region. The sampled animals were selected according to their known history or origin and predefined morphological characteristics (Table S1). The morphological characteristics of the animals were predefined following Atlas of farm animals in the Arab countries reported by ACSAD (The Arab Center for the Studies of Arid Zones Dry lands). (2011). The populations were then characterized by their distinctive phenotypes as can be seen from Table S1. For example, Najdi sheep is tall and black coated color with white face, have Roman nose and dropping ears and silky hair. Naemi and Awassi are brown face and white-skinned sheep, whereas Harri and Rufidi are white face and white-skinned sheep. On the other hand, Arb is black body color. All studied populations are fat-tail sheep (ACSAD (The Arab Center for the Studies of Arid Zones Dry lands)., 2011).

The blood sampling and animals handling were practiced with the permission of and in accordance with the guidelines of the Ethics Committee of King Saud University and Saudi Arabia National Committee of Bio Ethics (No. RG-1435-064). Blood sampling was performed by taking 5 ml of blood out of Jugular vein into EDTA tubes. The tubes were stored immediately in Ice-boxes and shortly after they were stored at −20°C until DNA extraction step was performed.

### DNA extraction and genotyping

Genomic DNA was extracted from 1 mL blood aliquots using commercially available DNA extraction Kit (E.Z.N.A® MicroElute Genomic DNA extraction Kit; OMEGA-Bio-Teck, 2010). DNA concentrations and purity were determined and then all samples were standardized to 10 ng/μL for genotyping process. Nineteen microsatellite (MS) markers recommended by the FAO/ISAG Panel (FAO, 2011), were used for genotyping purposes (Table 1). The MS markers are highly polymorphic microsatellite markers, which are short sequence repeats of 1–6 base pairs (FAO, 2011). The genotyping thermal cycling reaction was, in brief, performed on a GeneAmp® PCR system 9700; Applied Biosystem. The PCR cocktail was made in a volume of 10 μL. The amplification conditions were an initial denaturation cycle of 5 min at 94°C followed by the denaturation step at 95°C for 45 s. Then annealing step was immediately performed at recommended temperature of each primer for 1 min followed by final temperature as extension step at 72°C for 1 min. They were repeated for amplification. Then a final extension step at 72°C for 10 min was included. The Amplified PCR products were fragmented using 3130 Genetic Analyzer of Applied Biosystem Company®. The size of the microsatellite alleles was scored using Gene Mapper software®.

**Table 1:** Number of alleles (*NA*), Allelic Richness (*AR*), expected heterozygosity(*H*_*e*_), and inbreeding coefficient (*F*_*is*_) at each microsatellite locus and breed.

**Locus ID**	**Allele number**						**Allelic richness**							**Expected heterozygosity**						***F***_***IS***_					
	**Harri**	**Najdi**	**Naemi**	**Awassi**	**Arb**	**Rufidi**	**Harri**	**Najdi**	**Naemi**	**Awassi**	**Arb**	**Rufidi**	**Mean**	**Harri**	**Najdi**	**Naemi**	**Awassi**	**Arb**	**Rufidi**	**Harri**	**Najdi**	**Naemi**	**Awassi**	**Arb**	**Rufidi**
*MCM42*	8	6	6	5	7	4	2.6	2.5	2.3	2.8	2.5	2.9	2.5	0.71	0.67	0.61	0.76	0.67	0.8	−0.02	−0.02	0.24	−0.11	−0.1	0
*OARFCB20*	12	13	11	7	8	5	3.2	3.4	3.3	3.5	3.2	2.6	3.4	0.85	0.89	0.87	0.92	0.85	0.67	0.02	−0.02	−0.05	0.11	0.06	0.11
*OARVH72*	8	8	7	5	7	6	2.7	2.7	2.8	2.8	2.3	3.2	2.7	0.72	0.72	0.75	0.76	0.61	0.84	0.1	0.08	−0.16	−0.11	−0.02	0.06
*TGLA53*	11	10	10	7	9	4	3.4	3.2	3.4	3.5	2.8	2.7	3.3	0.89	0.84	0.88	0.91	0.74	0.73	0.06	0.07	0.14	0.09	0.06	0.2
*DYMS1*	10	12	16	8	10	3	3.3	3.3	3.3	3.6	3.1	2.3	3.3	0.87	0.88	0.86	0.92	0.81	0.64	0.24	0.25	0.13	0.11	0.17	0.71
*ILSTS044*	14	14	11	6	12	7	3.4	3.3	3.0	3.3	3.2	3.5	3.3	0.88	0.87	0.81	0.88	0.85	0.91	0.03	0.12	0.24	0.45	0.06	−0.11
*ILSTS05*	10	9	9	4	4	3	3.0	2.9	2.9	2.9	2.5	3.0	2.9	0.81	0.78	0.78	0.79	0.68	0.83	0.28	0.11	0.1	0.05	0.36	0.5
*MAF209*	10	9	8	5	5	4	3.0	3.0	3.0	2.9	2.8	2.7	3.0	0.8	0.81	0.79	0.79	0.74	0.73	0.09	0.16	0.25	−0.06	0.03	0.2
*BM8125*	5	6	7	7	4	3	2.4	2.3	2.1	3.4	2.4	1.8	2.4	0.64	0.61	0.53	0.89	0.63	0.38	0.2	0.06	0.03	0.07	−0.03	−0.07
*MAF214*	2	2	3	2	2	2	1.9	1.8	1.9	1.9	1.8	2.0	1.9	0.51	0.45	0.52	0.53	0.44	0.57	0.7	0.54	0.44	0.71	0.44	0.91
*OARFCB11*	10	10	11	6	7	4	3.1	3.3	3.2	3.3	3.0	2.9	3.2	0.82	0.86	0.85	0.88	0.8	0.8	0.08	0.04	0.12	−0.15	0.03	0
*OARJMP29*	10	10	8	6	8	4	3.0	2.9	2.8	3.2	2.8	3.0	2.9	0.8	0.78	0.76	0.85	0.76	0.82	0.12	0.16	0.04	0.23	0.04	0.1
*HUJ616*	8	7	9	5	9	3	2.8	2.5	2.7	2.7	2.9	2.1	2.8	0.76	0.66	0.74	0.73	0.78	0.51	−0.07	0.01	−0.17	−0.16	−0.19	−0.2
*OARFCB226*	13	14	11	7	9	7	3.4	3.3	2.6	3.2	3.2	3.6	3.2	0.9	0.86	0.66	0.83	0.84	0.93	0.01	0.06	0.09	0	0.05	−0.08
*SRCRSP09*	5	5	6	5	7	4	2.8	2.7	2.5	3.0	2.4	2.7	2.7	0.75	0.73	0.69	0.8	0.62	0.73	0.03	−0.05	−0.33	−0.29	−0.2	0.2
*BM1329*	9	9	8	4	6	4	2.3	2.2	2.6	2.8	2.5	2.8	2.5	0.6	0.51	0.69	0.76	0.68	0.78	0.27	0.48	0.36	0.13	0.23	0.25
*HSC*	14	13	11	6	12	5	3.5	3.4	2.9	3.3	3.3	3.1	3.4	0.91	0.88	0.79	0.86	0.86	0.84	0.06	−0.04	0.15	0.04	−0.01	0.06
*OARHH47*	11	12	10	5	10	5	3.2	3.3	3.3	2.8	3.3	3.2	3.4	0.84	0.87	0.87	0.74	0.88	0.86	−0.02	0.08	−0.02	0.11	−0.05	0.14
*SRCRSP5*	3	3	6	3	3	3	2.3	2.2	2.5	2.1	1.9	2.4	2.4	0.64	0.58	0.67	0.53	0.5	0.68	0.31	0.3	0.51	0.39	0.6	0.29
*Mean*	9.1	9.1	8.8	5.4	7.3	4.2	2.9	2.9	2.8	3.0	2.7	2.8	2.9	0.77	0.75	0.74	0.8	0.73	0.74	0.096	0.091	0.077	0.057	0.045	0.095
*s.d*.	3.4	3.6	2.8	1.5	2.8	1.4	0.45	0.49	0.42	0.45	0.45	0.48	0.43	0.11	0.14	0.11	0.11	0.12	0.14	*P* < 0.0001	0.0001	0.001	0.197	0.026	0.13

### Analyses of genetic diversity and structure

The number of alleles (*A*), allelic richness (*AR*), and expected heterozygosity (*H*_*e*_) for each locus and breed were estimated using FSTAT software (Goudet, 1995). Estimating *A* is complicated by the effects of sample size where large samples are expected to have more alleles. In order to correct estimates of *A* for differences in sample sizes of the studied populations, the estimates of *AR* were taken into account in order to overcome any possible bias resulting from the variation in sample sizes (Kalinowski, 2004). The small sample sizes of Awassi and Rufidi breeds were specifically reconsidered in AR analysis. Analysis of molecular variance (AMOVA) including coefficients of *F*-statistics, pairwise differentiation coefficient (*F*_*st*_) and intra-population differentiation (*F*_*is*_) (Hedrick, 2000), were computed under Hardy-Weinberg equilibrium (*HWE*) (Nei, 1987) using ARLEQUIN Software (Excoffier et al., 2005). Population structure was analyzed using STRUCTURE (Version 2.3.3) software (Pritchard et al., 2000) considering an admixture model with correlated allele frequencies between breeds. The length of the burn-in and Monte Carlo Markov chain (MCMC) simulations were 200,000 and 100,000, respectively, in 50 runs for each number of clusters (*K*) ranging between 2 and 6. The *K*-value, log probability of the data (L[K]) values for each cluster were estimated. The results were exported to STRUCTURE HARVESTER (Earl and von Holdt, 2012) for plotting the likelihood membership coefficient (*DeltaK*) values so as to determine the most likely number of clusters. Finally, GENETIX® software was used to perform factorial correspondence analysis (Belkhir et al., 2000). The factorial correspondence analysis is a multidimensional statistical method to evaluate the number of genetic groups (Belkhir et al., 2000).

## Results

### Genetic diversity

The results of within-population genetic variation were based on the values of allelic (*A* and *AR*) and genetic diversity (*H*_*e*_) (Table 1). The mean *A* was 9.1, 9.1, 8.8, 5.4, 7.3, and 4.2 for Harri, Najdi, Naemi, Awassi, Arb, and Rufidi breeds, respectively. In general, the majority of alleles were found in all the breeds, except those in Awassi and Rufidi breeds in which small sample sizes might explain the comparatively small mean *A*. The *A* per breed ranged from 2 in all breeds, except Naemi (*A* = 3), to 14 in both the Harri and the Najdi breeds (Table 1). At the loci level, the lowest number of *A* = 2 was found at locus *MAF214* in all the breeds except Naemi, whereas the highest number of *A* = 14 was found at the loci *ILSTS044* in the Harri and the Najdi, and at *OARFCB226* in the Najdi and at *HSC* in the Harri breed. Both the Harri and the Najdi had the highest and similar *A* at most of the studied loci along with the same value of mean *A* (Table 1). The result might indicate that both breeds have a similar genetic background. The mean *AR*-values were 2.9, 2.9, 2.8, 3.0, 2.7, 2.8, and 2.9 for Harri, Najdi, Naemi, Awassi, Arb, and Rufidi breeds, respectively. The average *AR* per breed was the lowest (2.7) in the Arb and highest (3.0) in Awassi sheep (Table 1). It is notable that the average *AR-*value for both Harri and Najdi was the same as was observed for their *NA*. The lowest *AR* = 1.8 was found at locus *MAF214* in Arb sheep and at *BM8125* in Najdi sheep, whereas the highest *AR* = 3.6 was observed at the *DYMS1* in Awassi breed and *OARFCB226* in the Rufidi sheep (Table 1).

The average *H*_*e*_was 0.77, 0.75, 0.74, 0.80, 0.73, and 0.74 for the Harri, Najdi, Naemi, Awassi, Arb, and Rufidi breeds, respectively (Table 1). The results showed slightly higher *H*_*e*_in the Awassi sheep over the values of the other breeds (Table 1) as was the value of *AR*. Overall, the average *H*_*e*_at the 19 MS loci ranged from 0.73 to 0.80, reflecting a small range of differences between values for the breeds and indicating high genetic variation. On the other hand, it shows that the Arb breed had the lowest *H*_*e*_, but still at least 73%. The AMOVA showed that the extent of genetic variation was 2.79, 7.85, and 89.45% between the breeds, among the individuals within the breeds and within the individuals, respectively (Table S2).

The AMOVA results also showed a significant positive inbreeding coefficient (*F*_*is*_) indicating less heterozygosity than it is expected under *HWE* in four sheep breeds; 0.096, 0.091, 0.077, and 0.045 (*P* < 0.002, 0.001, 0.002, and 0.027) for Harri, Najdi, Naemi, and Arb, respectively (Table 1). The values of *F*_*is*_for the other breeds-Awassi and Rufidi-were not significant (Table 1). The *F*_*is*_-values at the loci varied from -0.33 at *SCRCRSP09* in the Naemi to 0.91 at *MAF14* in the Rufidi breed. On the other hand, seven MS loci (*TGLA53, DYMS1, ILSTS05, MAF214, OARJMP29, BM1329*, and *SRCRSP5*) showed positive *F*_*is*_*-*values in all the breeds. The results indicated a shortage of heterozygotes than it would be expected under HWE. The remaining loci showed that the *F*_*is*_*-*value was either negative or positive in one breed or more (Table 1).

**Figure 2:**
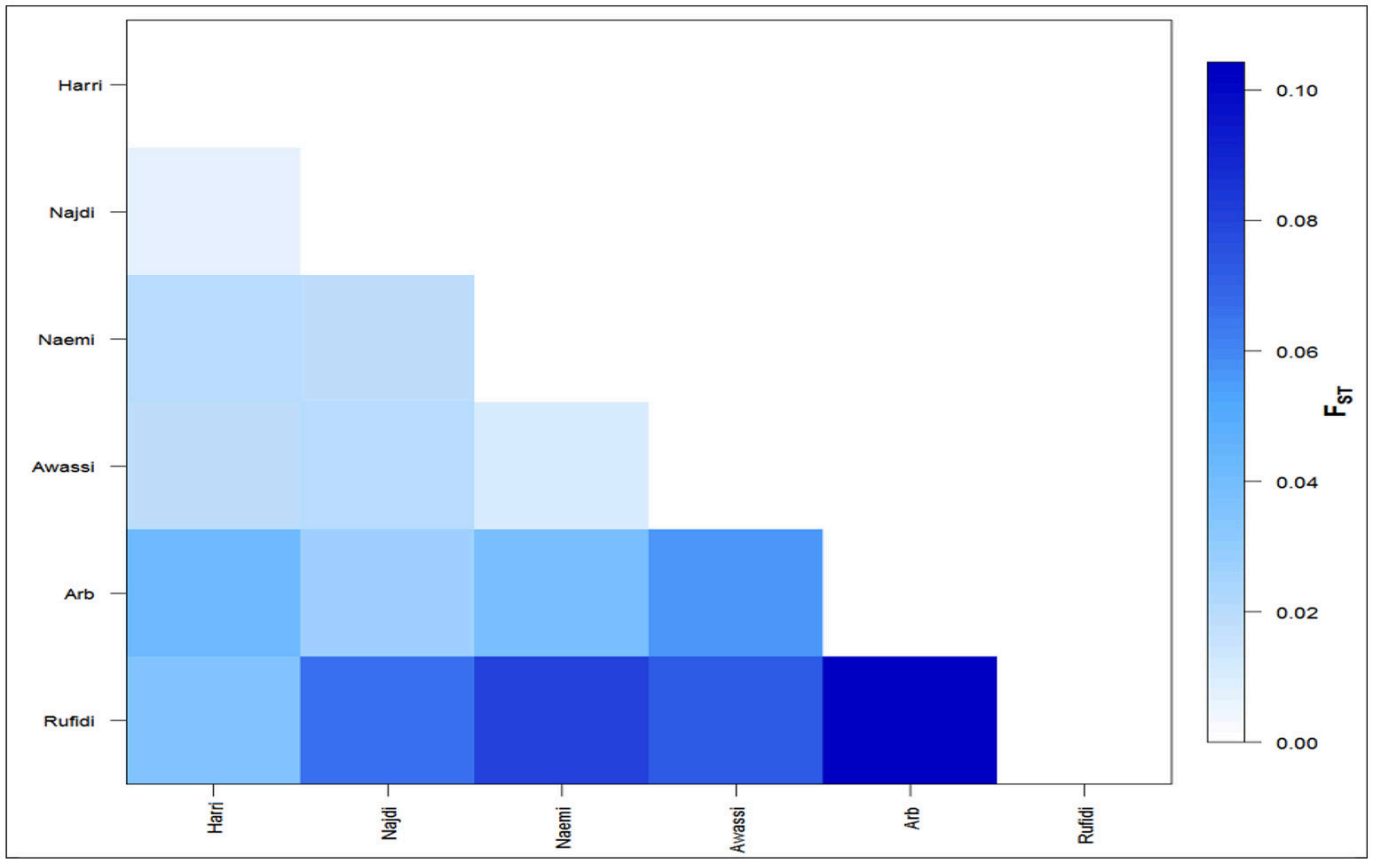
The average number of pairwise differences (*Fst*) between the Arabian sheep breeds.

The differentiation coefficients (*F*_*st*_) based on the distance method of different allele numbers were found significant between pairwise comparisons except between Naemi and Awassi. The pairwise *F*_*st*_-values varied from lowest (0.006) between Neami and Awassi to highest (0.104) between the Arb and the Rufidi breeds (Figure 2). The *F*_*st*_-values showed a high differentiation coefficient between Rufidi with the other breeds. The next highest level of differentiation was between Arb and the other populations. Furthermore, Figure 2 shows a lower differentiation between the Harri and the Najdi, while a higher differentiation was observed between the Harri and the Arb. The lowest genetic differentiation was observed between Awassi and Najdi and the rest of the other populations, respectively.

### Genetic population structure

**Figure 3:**
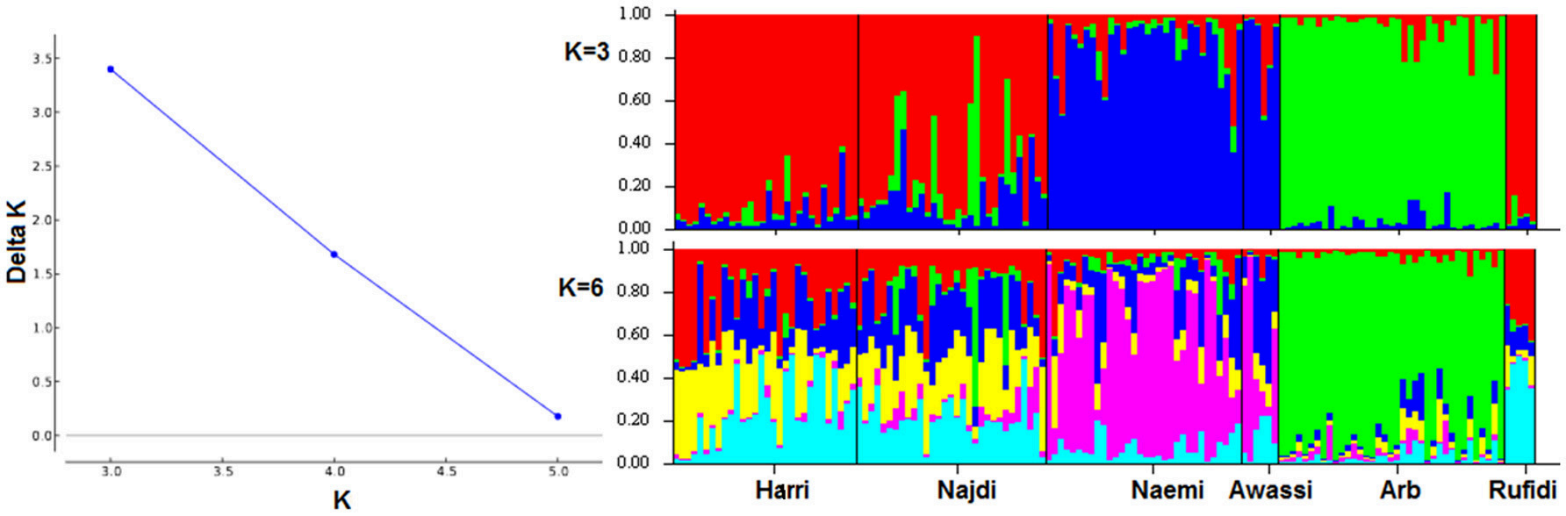
**(A)** Plot of Delta *K*-values for each *K* from 3 to 6 (**B)**. Estimated proportion of membership for each individual represented by a single vertical line broken into *K* colored segments, with lengths proportional to each predefined cluster of *K* from 2 to 6.

The genetic population structure of each breed was determined based on admixture level for each sheep individual using correlated allele frequencies model implemented within the *STRUCTURE* software. The results of Delta K indicated that the optimal number of genetic clusters representing most like ancestral breeds was at *K* = 3 (Figure 3A). The value suggests that the studied sheep breeds were better defined by three genetic clusters/backgrounds instead of six breeds (Figure 3). The three clusters/genetic backgrounds were made up of Harri, Najdi, and Rufidi in the first, Naemi and Awassi in the second and Arb in the third cluster (Figure 3). In Figure 3, each individual is represented by a single vertical line broken into *K* colored segments (Figure 3). The mixed colors with proportional lengths represent the admixture level for predefined populations of *K* between 3 and 6. The first genetic pool had individuals of Harri, Najdi, and Rufidi sheep with different assignment probabilities (~60%) (Figure 3). Similarly, many individuals of this gene pool have a reasonable color broken proportion with blue color mainly. Some individuals of Najdi had high assignment probabilities with the second cluster (Naemi and Awassi). Worth noting was that the Najdi had a good proportion of admixture in its individuals from the second and third genetic pools. The few individuals were shown with broken colors with green color in probabilities (~70%). It might be better to consider it from the second cluster instead. Alternatively, most individuals of the second pool (Blue color; *K* = 3) were solely assigned Naemi and Awassi together. The third genetic pool had Arb breed with very few individuals of limited admixture proportion (<20%) of the second gene pool. The shared proportion of the second gene pool was observed in the other two pools, indicating a common ancestry origin.

### Correspondence analysis

**Figure 4:**
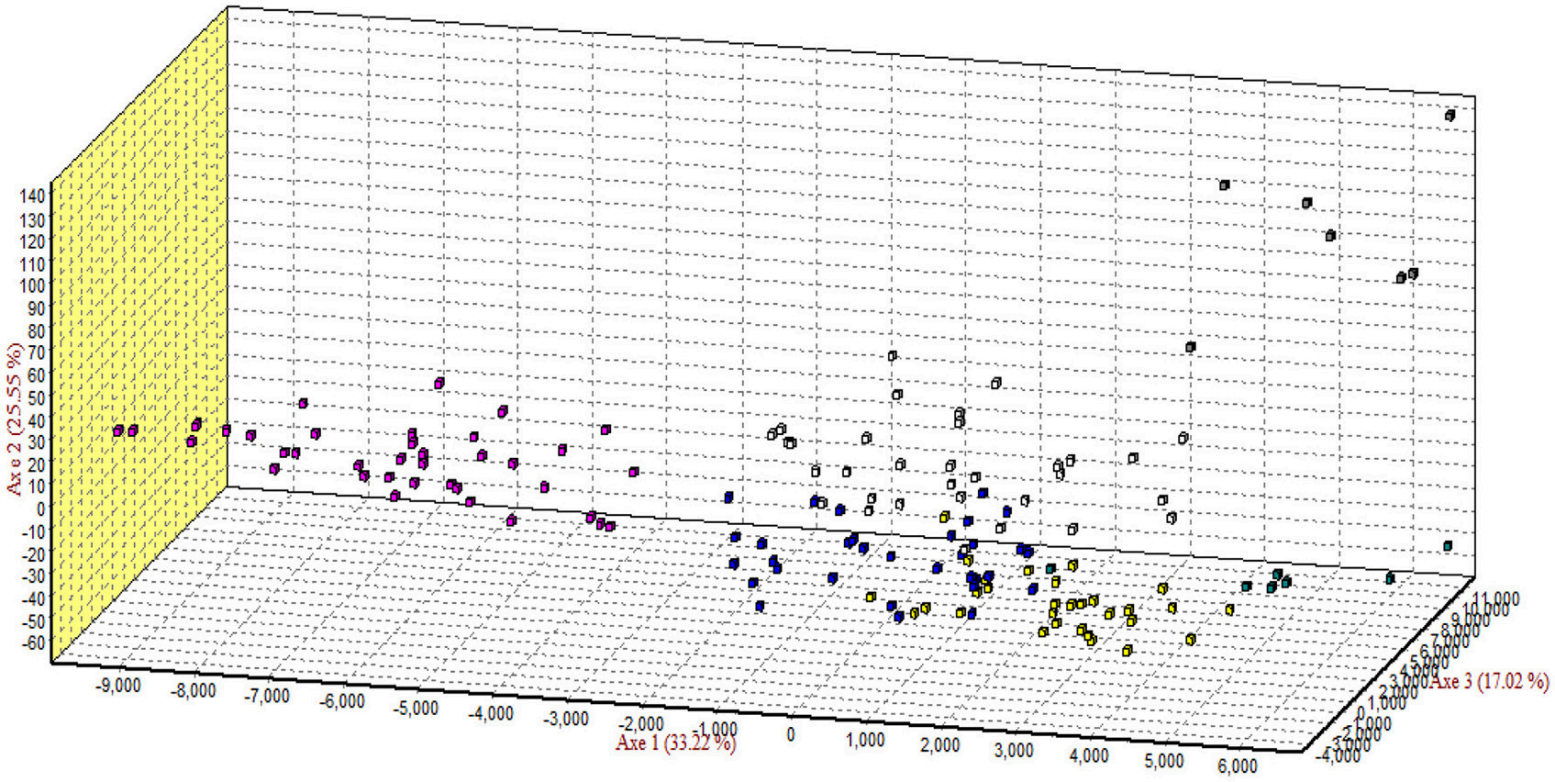
Correspondence analysis of the genotypes of all individuals of the breeds. Square colors: Rufidi 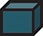, Harri 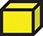, Najdi 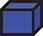, Naemi 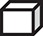, Awassi 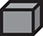 and Arb 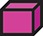.

The results of correspondence analysis in this study highlighted better genetic admixture and differentiation between all individuals within and between the breeds (Figure 4). The results are represented in three factorial dimensional graphs where the first, second, and third factors (axes 1, 2, and 3) accounted for 33.92, 25.39, and 17.02% of total variation, respectively. The analysis clearly distinguished Arb individuals from those of the other breeds. Furthermore, Awassi individuals were more distinguished from the other breeds, but closer to Naemi (Figure 3). Most of the individuals clustered into groups that belonged to each predefined breed rather than being in mixed populations. However, Harri and Najdi individuals showed admixture as was observed in the structure analysis.

## Discussion

The KSA imports millions of sheep every year for local consumption and sacrifice during the Eid Al-Adha religious festival. The animal importation represents a current animal exchange networks and span countries as far as Australia and nearby ones such as those of the Horn of Africa, Yemen, Gulf, and Middle East. Consequently, the genetic structure of indigenous sheep in The KSA could have been influenced by demographic events such as animal exchange network imbedding gene flow. Indeed, the main question driving our study was whether the genetic structure of KSA sheep was influenced more by internal gene flow, breeding practices and geographical features. The high genetic variation observed within and between sheep breeds indicated by the *A, RA* and *H*_*e*_could be the result of one or more past evolutionary events. The most likely reason to explain the high genetic variation, considering that the transhumant system still predominates in all regions of KSA, was gene flow. This reason could have involved past gene flow within the breeds reared in the same and adjacent regions. The best evidence for this occurrence is reflected in the individuals of Harri and Najdi breeds which existed in the same flocks reared from South to central regions with many crossbreds. The *A* and *H*_*e*_*-*values were high at most of the loci studied in all the breeds. In particular, the Harri and Najdi had the highest *A* as well as *H*_e_ indicating that they are the most genetically variable breeds. Generally, if recipient populations have different allele frequencies and if selection is not operating, then it might be expected that migration alone would rapidly cause genetic variation (Ridley, 2004). Our finding shows that the Awassi breed was the most varied breed. Earlier reports showed that *H*_*e*_was 0.696 for Jordan Awassi (Al-Atiyat, 2015) and 0.667 for the Turkey Awassi (Soysal et al., 2005). It seems that high values of *H*_*e*_ were not uncommon in the Awassi sheep, the most common breed in the Middle East.

The overall *F*_*is*_*-*value for each breed was positive, indicating a certain level of heterozygote deficiency. The positive *F*_*is*_-values indicate that individuals in a population are more related than expected under a model of random mating and suggest that the sheep breeds had higher value of inbreeding. This could be due to small population sizes, selection pressure and population subdivision (Hedrick, 2000). The latter can be explained as a Wahlund effect which is reduction in the heterozygosity as a result of population subdivision (Hedrick, 2000). The Wahlund effect: the same situation can be used to characterize the Naemi sheep which subdivided into different population across several regions. On the other hand, the heterozygote deficiency may be due to the fact that a small number of breeding males are used in mating or in the last few decades mating had been occurring among closely related animals. This is observed mainly in Najdi and Harri flocks. Even though gene flow was noticed into these two breeds, it was not enough to drive the individuals into excess of the heterozygosity. The lowest *F*_*st*_*-*value between these two breeds provided extra proof that they are closely genetically related. In a wider study, sheep of the world were found to be differentiated on the national and international levels (Kijas et al., 2012). For instance, Awassi from different countries were highly differentiated from the Australian Merino sheep (Al-Atiyat et al., 2014), the Spanish Merino (Arranz et al., 1998), Turkish sheep (Ozdemir et al., 2011) and the Middle East fat-tailed sheep (Rocha et al., 2011) and Egyptian sheep (Elbeltagy et al., 2015).

Structure and admixture analyses have been used in earlier studies involving different sheep populations, providing an appropriate approach to determine ancestral, pure and hybrid populations (Alvarez et al., 2004; Ligda et al., 2009). Although the results of STRUCTURE showed admixture at the individual level in each sheep breed, the six breeds could be clustered into three gene pools. All individuals of the Arb sheep were assigned to a separate gene pool, with few individuals showing a small fraction of admixture deriving from a common ancestry (Figure 3). The results also showed at the individual level that Naemi and Awassi had a mixed ancestry as a result of sharing a fraction of their genome inherited from ancestors; whereas it is much less for individuals of the other breeds. The results might need further justification to prove the observed integration related to breeding practices, geographical isolation and/or common ancestry. It is widely accepted that world's sheep breeds reflect high levels of historical admixture and strong recent selection (Kijas et al., 2012). On the other hand, the clear admixture proportion found between Najdi and the gene pool of both Naemi and Awassi reflect possibly shared ancestry and past individual migration in the same geographical regions. In fact, looking back to the sampling regions where these four breeds (Harri, Najdi, Naemi, and Awassi) came from, we found that these regions were considered to be the major livestock husbandry region where transhumant production system was common and recent crossing observed. The observed genetic structure might be related to the geographical features of the region from which the breeds were sampled. This result was probably, first of all, due to shared ancestry and second due to gene flow between the populations being reared in the close geographic areas. Nevertheless, Arb sheep was geographically isolated in the East region of the KSA with very limited dispersals across the other regions of KSA. The indigenous nomadic people were extremely in favor of practicing pure breeding of the breed and objecting to any crossbreeding strategy. Therefore, the genetic structure of Arb sheep could be influenced by founder effect because they have been isolated in geographical confines in the East region of KSA. As a consequence, the graphic representation of correspondence analysis (Figure 3) showed a clear separation of Arb individuals. Clearly the study populations are subdivided into three groups matching the results of structure analysis. Despite the fact that the Awassi sheep breed was located far from those studied groups, they are definitely closer to Naemi sheep. The three groups were matched to their geographic distribution of their sampling locations. These results are in agreement with known history of the breeds in regard to their geographic locations and their long evolutionary history associated with past common ancestors. In general, the result was similar to previous findings which showed close genetic relationship between the four KSA sheep breeds (Aljumaah et al., 2014) and the native Jordan Awassi sheep (Al-Atiyat et al., 2014; Al-Atiyat, 2015). Furthermore, Turkish Awassi sheep as a fat-tail sheep was separated from other Turkish sheep breeds based on correspondence analysis (Ozdemir et al., 2011). Indeed, the Near East region is considered to be the main center of origin of specifically the fat-tailed sheep (Rocha et al., 2011). In agreement, the Jordan Awassi shows no common genetic structure with the Australian Merino most likely due to geographic isolation (Al-Atiyat, 2015). On the other hand, evidence of gene exchange between Egyptian sheep breeds was reported for flocks reared in the same region (Elbeltagy et al., 2015). Furthermore, Kijas et al. (2012) reported that World's sheep breeds reveal high levels of historic admixture and strong recent selection.

## Conclusion

The sheep breeds of the KSA revealed high genetic diversity considering that they are reared in different geographic regions that are far apart and with different features. The Arb sheep was the most differentiated breed, whereas Jordan Awassi was least differentiated from Naemi sheep indicating their common ancestry. The population structure analysis identified three main gene pools underlying the ancestral genetic diversity. The first had Harri, Najd, and Rufidi, the second had Neami and Awassi, whereas the third pool had Arb breed. In accordance, the factorial correspondence analysis distributed the individuals in the three genetic groups. The resulted genetic structure of all gene pools had limited shared genetic makeup arising from common ancestry. Furthermore, the first and the second gene pools could have arisen from past and recent gene flow between individuals. The gene flow was evident between different flocks rearing two or more breeds under transhumant production system. The third pool might have resulted from geographical separation/isolation. These results are in agreement with known history of the breeds in regard to their geographical location and their expected common evolutionary history.

## Ethics statement

Standard techniques were used to collect blood. The procedure was reviewed and approved by the University of Edinburgh Ethics Committee (reference number OS 03-06) and also by the Institute Animal Care and Use Committee of the International Livestock Research Institute, Nairobi.

## Author contributions

RA-A and RA conceived and designed the experiment. RA and MA performed the experiment. RA-A and RA analyzed the data. RA-A performed the bioinformatics analysis. MA and AA contributed in data. RA-A, RA, and AA wrote the manuscript. All authors have agreed on the contents of the manuscript.

### Conflict of interest statement

The authors declare that the research was conducted in the absence of any commercial or financial relationships that could be construed as a potential conflict of interest.
