# Supplementary material for: Microsatellite-Based Genetic Structure and Diversity of Local Arabian Sheep Breeds
Source: Front Genet. 2018 Sep 25;9:408. doi: 10.3389/fgene.2018.00408 (PMC6167516; doi:10.3389/fgene.2018.00408)
Supplement: Supplementary file 1 [file Table_1.DOCX]

**Table S1.** Representative images of the six sheep breeds sampled in selected flocks. The images are representing a group and an individual adult from the breed in a flock it was sampled from.

| 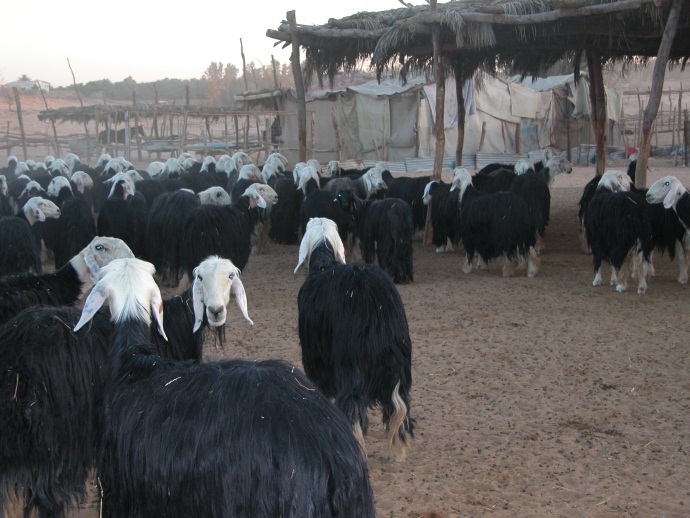 | 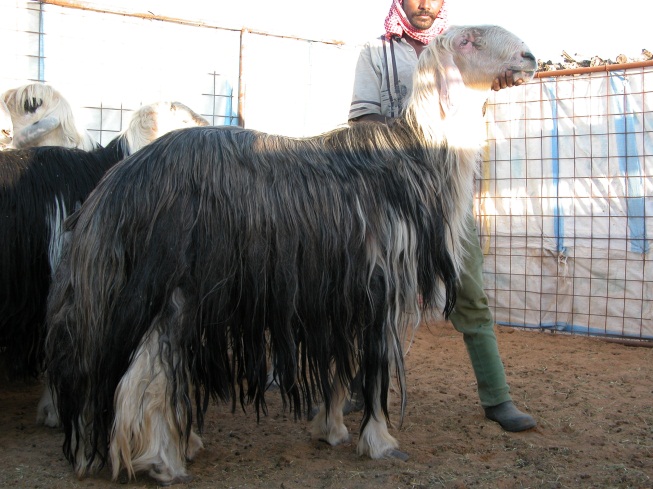 |
| --- | --- |
| Najdi flock | Adult Najdi male |
| 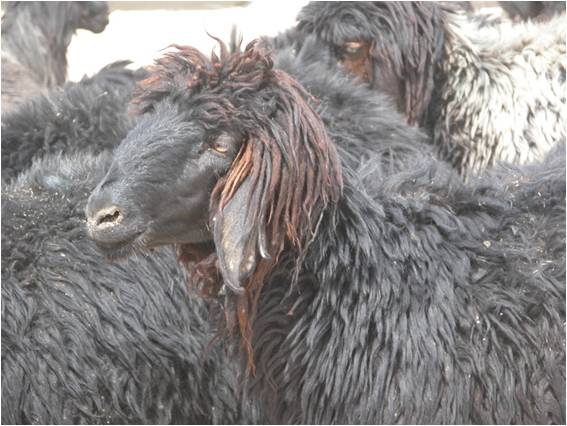 | 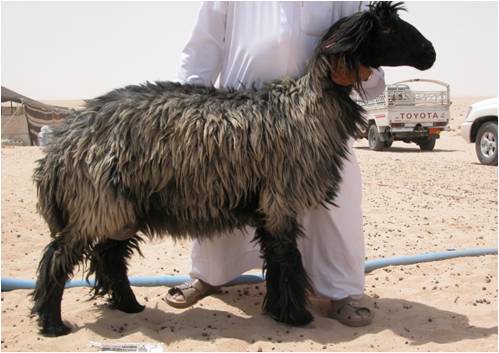 |
| Arb flock | Adult Arb female |
| 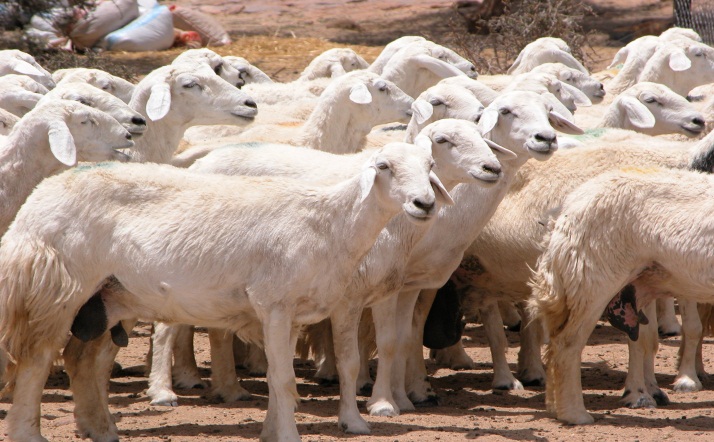 | 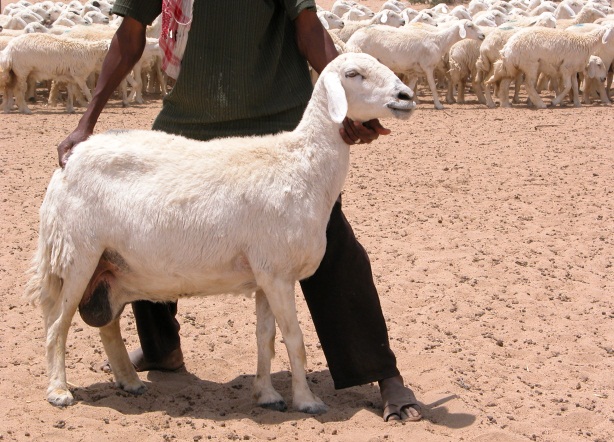 |
| Harri flock | Adult Harri female |
| 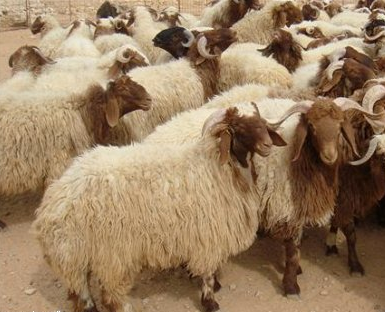 | 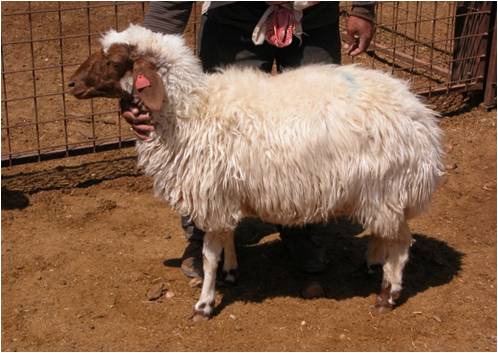 |
| Naemi flock | Adult Naemi male |
| 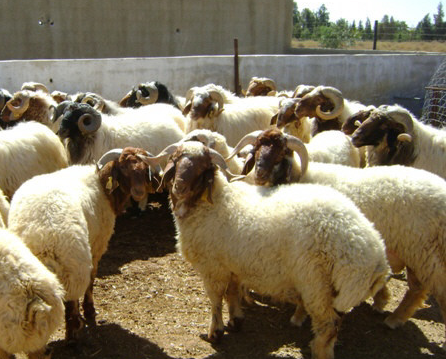 | 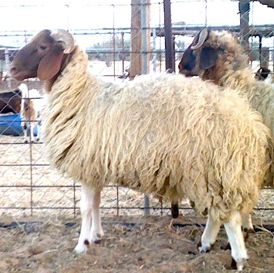 |
| Awassi flock | Adult Awassi male |
| 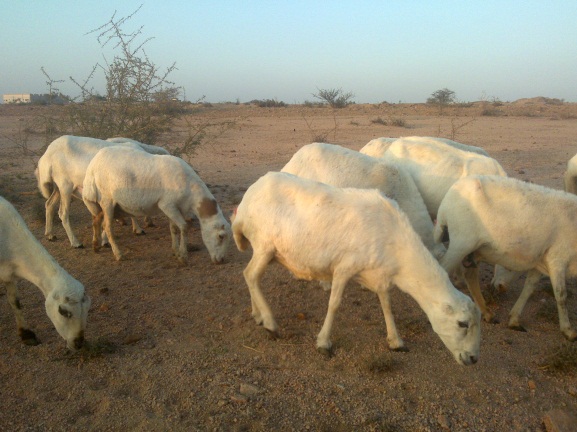 | 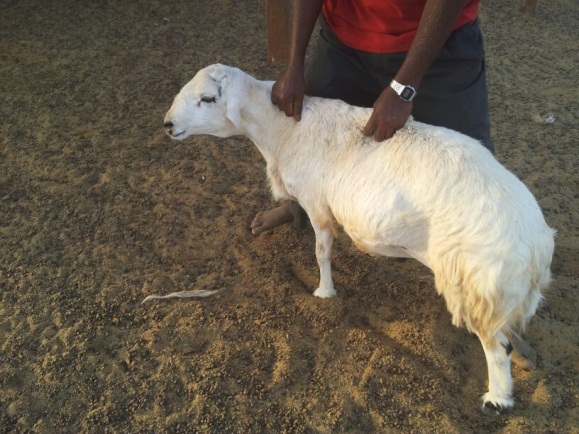 |
| Rufidi flock | Adult Rufidi female |
