# Supplementary material for: Microsatellite-Based Genetic Structure and Diversity of Local Arabian Sheep Breeds
Source: Front Genet. 2018 Sep 25;9:408. doi: 10.3389/fgene.2018.00408 (PMC6167516; doi:10.3389/fgene.2018.00408)
Supplement: Supplementary file 2 [file Table_2.docx]

**Table S2.** . List of genotyped microsatellite primer names, their sequences of forward and reverse (5’-3’), Annealing temp.(°C), Gene Bank Accession number, and predicted product sizes (in brackets).

|  | **Name(s)** | **spec** | **Chromosome** | **Primer sequence (5’-3’)**  Forward Reverse | | **Annealing temp.(°C)** | **Genebank accession number** | **Allele range (bp)** |
| --- | --- | --- | --- | --- | --- | --- | --- | --- |
|  | OarHH47 | OAR | 18 | TTTATTGACAAACTCTCTTCCTAACTCCACC | GTAGTTATTTAAAAAAATATCATACCTCTTAAGG | 58 | L12557 | 130-152 |
|  | OarVH72 | OAR | 25 | GGCCTCTCAAGGGGCAAGAGCAGG | CTCTAGAGGATCTGGAATGCAAAGCTC | 57 | L12548 | 121-145 |
|  | BM1329 | OAR | 6 | TTGTTTAGGCAAGTCCAAAGTC | AACACCGCAGCTTCATCC | 50 | G18422 | 160-182 |
|  | BM8125 | OAR | 17 | CTCTATCTGTGGAAAAGGTGGG | GGGGGTTAGACTTCAACATACG | 50 | G18475 | 110-130 |
|  | HUJ616 | OAR | 13 | TTCAAACTACACATTGACAGGG | GGACCTTTGGCAATGGAAGG | 54 | M88250 | 114-160 |
|  | DYMS1 | OAR | 20 | AACAACATCAAACAGTAAGAG | CATAGTAACAGATCTTCCTACA | 59 | ... | 159-211 |
|  | SRCRSP9 | CHI12 | 12 | AGAGGATCTGGAAATGGAATC | GCACTCTTTTCAGCCCTAATG | 55 | L22201 | 99-135 |
|  | OarCB226 | OAR | 2 | CTATATGTTGCCTTTCCCTTCCTGC | GTGAGTCCCATAGAGCATAAGCTC | 60 | L20006 | 119-153 |
|  | ILSTS5 | OAR | 7 | GGAAGCAATGAAATCTATAGCC | TGTTCTGTGAGTTTGTAAGC | 55 | L23481 | 174-218 |
|  | OARFCB11 |  |  | GGCCTGAACT | CACAAGTTGATATATCTATC | 67 | L01531 | 67-58 |
|  | SRCRSP5 | OAR | 18 | GGACTCTACCAACTGAGCTACAAG | GTTTCTTTGAAATGAAGCTAAAGCAATGC | 56 | L22197 | 126-158 |
|  | MAF214 | OAR | 16 | GGGTGATCTTAGGGAGGTTTTGGAGG | AATGCAGGAGATCTGAGGCAGGGACG | 58 | M88160 | 174-282 |
|  | OarFCB20 | OAR | 2 | AAATGTGTTTAAGATTCCATACAGTG | GGAAAACCCCCATATATACCTATAC | 56 | L20004 | 95-120 |
|  | OarJMP29 | OAR | 24 | GTATACACGTGGACACCGCTTTGTAC | GAAGTGGCAAGATTCAGAGGGGAAG | 56 | U30893 | 96-150 |
|  | MCM42 |  | 9 | CATCTTTCAAAAGAACTCCGAAAGTG | CTTGGAATCCTTCCTAACTTTCGG | 55 | L34281 |  |
|  | HSC |  | 20 | CTGCCAATGCAGAGACACAAGA | GTCTGTCTCCTGTCTTGTCATC | 65 | M90759 |  |
|  | ILSTS44 |  |  | AGTCACCCAAAAGTAACTGG | ACATGTTGTATTCCAAGTGC |  |  |  |
|  | MAF209 | OAR | 17 | GATCACAAAAAGTTGGATACAACCGTGG | TCATGCACTTAAGTATGTAGGATGCTG | 63 | M80358 | ... |
|  | TGLA53 |  | 16 | GCTTTCAGAAATAGTTTGCATTCA | ATCTTCACATGATATTACAGCAGA | 55 . | D16S3 | 143-191 |
